# The microwave bacteriome: biodiversity of domestic and laboratory microwave ovens

**DOI:** 10.3389/fmicb.2024.1395751

**Published:** 2024-08-08

**Authors:** Alba Iglesias, Lorena Martínez, Daniel Torrent, Manuel Porcar

**Affiliations:** ^1^Institute for Integrative Systems Biology (I2SysBio), University of Valencia-CSIC, Valencia, Spain; ^2^Darwin Bioprospecting Excellence S.L., Valencia, Spain

**Keywords:** microwave, 16S rRNA gene sequencing, taxonomic classification, radiation, desiccation, selective pressure

## Abstract

Microwaves have become an essential part of the modern kitchen, but their potential as a reservoir for bacterial colonization and the microbial composition within them remain largely unexplored. In this study, we investigated the bacterial communities in microwave ovens and compared the microbial composition of domestic microwaves, microwaves used in shared large spaces, and laboratory microwaves, using next-generation sequencing and culturing techniques. The microwave oven bacterial population was dominated by *Proteobacteria*, *Firmicutes*, *Actinobacteria*, and *Bacteroidetes*, similar to the bacterial composition of human skin. Comparison with other environments revealed that the bacterial composition of domestic microwaves was similar to that of kitchen surfaces, whereas laboratory microwaves had a higher abundance of taxa known for their ability to withstand microwave radiation, high temperatures and desiccation. These results suggest that different selective pressures, such as human contact, nutrient availability and radiation levels, may explain the differences observed between domestic and laboratory microwaves. Overall, this study provides valuable insights into microwave ovens bacterial communities and their potential biotechnological applications.

## 1 Introduction

Microorganisms that thrive in ecosystems characterized by extreme environmental conditions have been well studied to elucidate the evolutionary mechanisms that have favored their adaptation. Natural extreme environments represent an exceptional source of novel microbial species, as well as a source of novel secondary metabolites with biotechnological applications (Shu and Huang, 2022). However, one does not need to travel that far in search for extreme environments.

As a result of human activity and modernization, many different man-made artificial devices were built in the last century. Many studies have described the microbial populations present in highly anthropized artificial environments such as elevator buttons (Kandel et al., 2014), the underground (Gohli et al., 2019), and small electronic devices (Lax et al., 2015). Other works have unveiled that some man-made devices, machines, and appliances, despite being in constant contact with humans or human activities, have their own microecosystems with their own selective pressures and conserved microbiomes. This is the case, for example, of coffee machines (Vilanova et al., 2015) or dishwashers (Raghupathi et al., 2018).

Microwave irradiation has been used for decades to reduce the presence of microorganisms in food and extend food shelf life. The application of an electromagnetic wave in the range of 300 MHz to 300 GHz to a dielectric medium such as food, also known as microwave heating, generates heat to reach lethal temperatures that inactivate most microorganisms, such as *Escherichia coli*, *Enterococcus faecalis*, *Clostridium perfringens*, *Staphylococcus aureus*, *Salmonella* spp. and *Listeria* spp. (Woo et al., 2000; Kubo et al., 2020). Recent work has shown that cell inactivation is associated with deactivation of oxidation-regulating genes, DNA damage and increased permeability and disrupted integrity of cell membranes (Cao et al., 2018; Shaw et al., 2021). Despite this extensive characterization of the biological effects of microwave radiation on foodborne bacteria, to our knowledge there are no reports of microwaves as microbial niches, that is, environments where specific selective pressures (in this case, thermal shock, microwave radiation, and desiccation) can shape a specifically adapted microbiome.

In the present work, we describe the bacterial composition of 30 microwaves from different environments (domestic, domestic of shared use, and laboratory) to explore the intricacies of the microwave microbiome, with a particular focus on identifying variations based on usage patterns. The goal is to determine whether microwaves harbor a distinct microbiome shaped by prolonged exposure to microwave radiation, or whether their bacterial communities are influenced by food interactions and user habits.

## 2 Results

### 2.1 Strain collection

**Figure 1:**
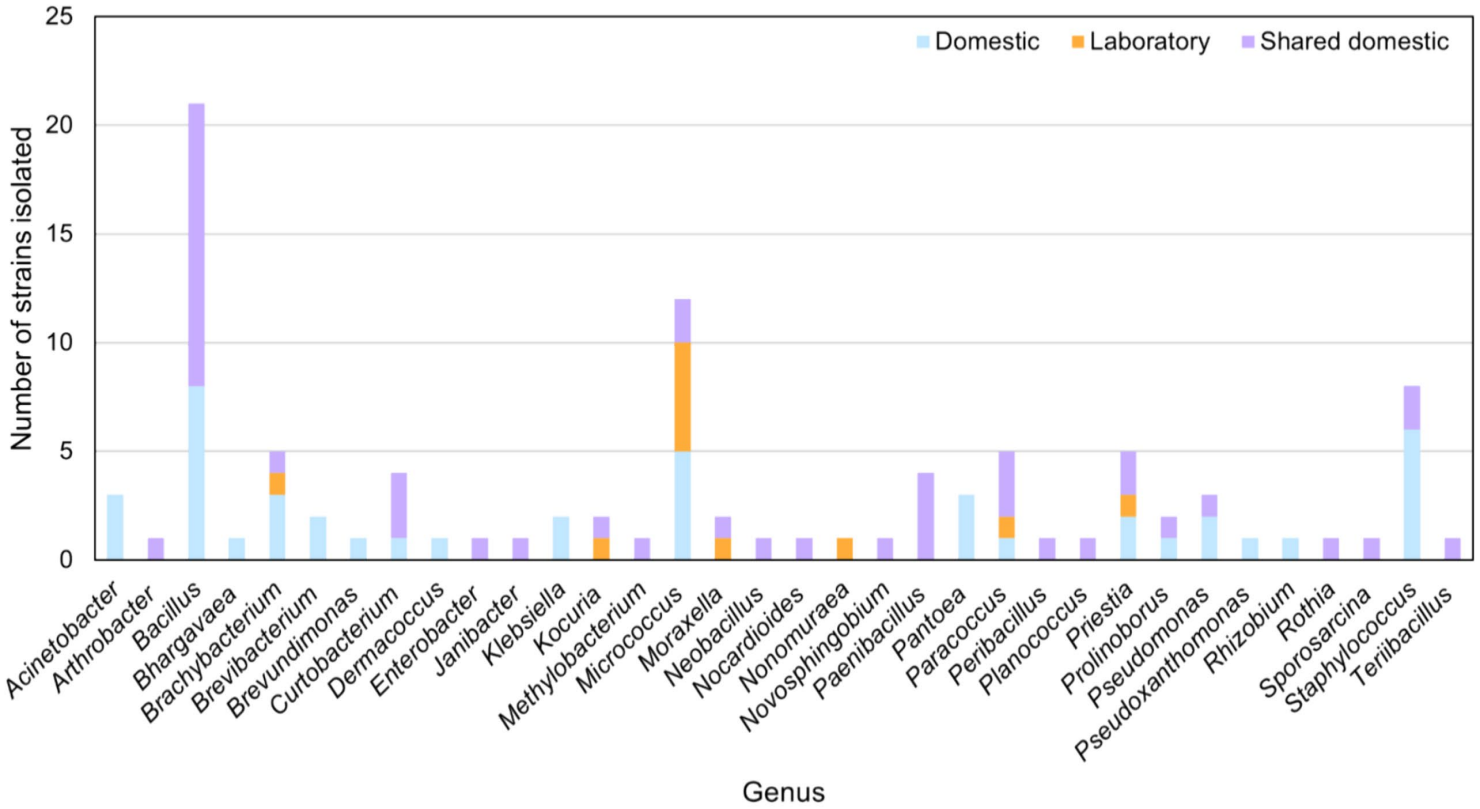
Main bacterial genera isolated from domestic, domestic-shared and laboratory microwaves.

Thirty microwave ovens (10 from domestic use, 10 of domestic shared-use, and 10 of laboratory use) were sampled and used to culture microbial strains on Columbia agar, TSA, YM, R2A, and NA. This yielded a collection of 101 isolates dominated by strains belonging to the genera *Bacillus*, *Micrococcus*, and *Staphylococcus*, followed by *Brachybacterium*, *Paracoccus*, and *Priestia*. Members of the genera *Acinetobacter*, *Bhargavaea*, *Brevibacterium*, *Brevundimonas*, *Dermacoccus*, *Klebsiella*, *Pantoea*, *Pseudoxanthomonas*, and *Rhizobium* were found only in domestic microwaves. Strains belonging to the genera *Arthrobacter*, *Enterobacter*, *Janibacter*, *Methylobacterium*, *Neobacillus*, *Nocardioides*, *Novosphingobium*, *Paenibacillus*, *Peribacillus*, *Planococcus*, *Rothia*, *Sporosarcina*, and *Terribacillus* were isolated only in microwaves of domestic-shared use. A strain of *Nonomuraea* species was isolated only in laboratory microwaves (Figure 1).

Moreover, microbial strains of the genera *Bacillus*, *Curtobacterium*, *Prolinoborus*, *Pseudomonas*, and *Staphylococus* were isolated from both domestic and domestic-shared microwaves. *Kocuria* and *Moraxella* strains were obtained from domestic-shared and laboratory microwaves. Members of four genera were found in all types of microwaves: *Brachybacterium*, *Micrococcus*, *Paracoccus*, and *Priestia* (Figure 1).

### 2.2 Analysis of bacterial diversity of microwaves by NGS

**Figure 2:**
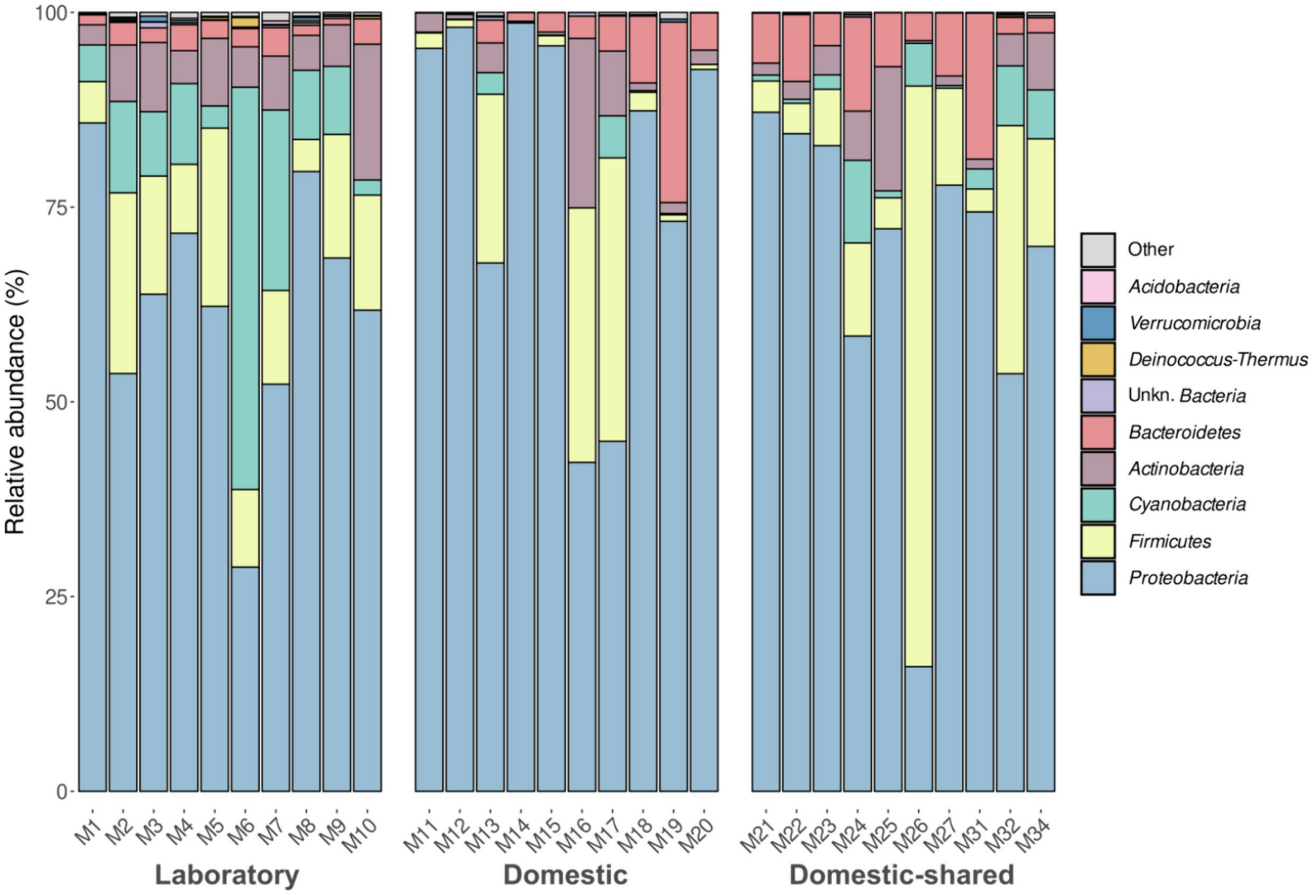
Taxonomic distribution at the phylum level of the bacteria present in the three types of microwaves: laboratory (M1–M10), domestic (M11–M20), and domestic-shared (M21–M34).

NGS (Next Generation Sequencing) analysis of the conserved V3 and V4 regions of the 16S rRNA gene allowed the exploration of bacterial diversity within microwave ovens. The results showed that, at the phylum level, *Proteobacteria* predominated in microwave bacterial communities, followed by *Firmicutes* and *Actinobacteria* to a lesser extent (Figure 2; Supplementary file S1). Differential abundance analysis confirmed the higher presence of the phyla *Chloroflexi*, *Acidobacteria*, *Deinococcus-Thermus*, and *Cyanobacteria* in the laboratory microwaves compared to the household microwaves (Supplementary file S2). The latter phylum was also more abundant in the domestic-shared microwave group compared to the domestic (not shared) microwaves.

**Figure 3:**
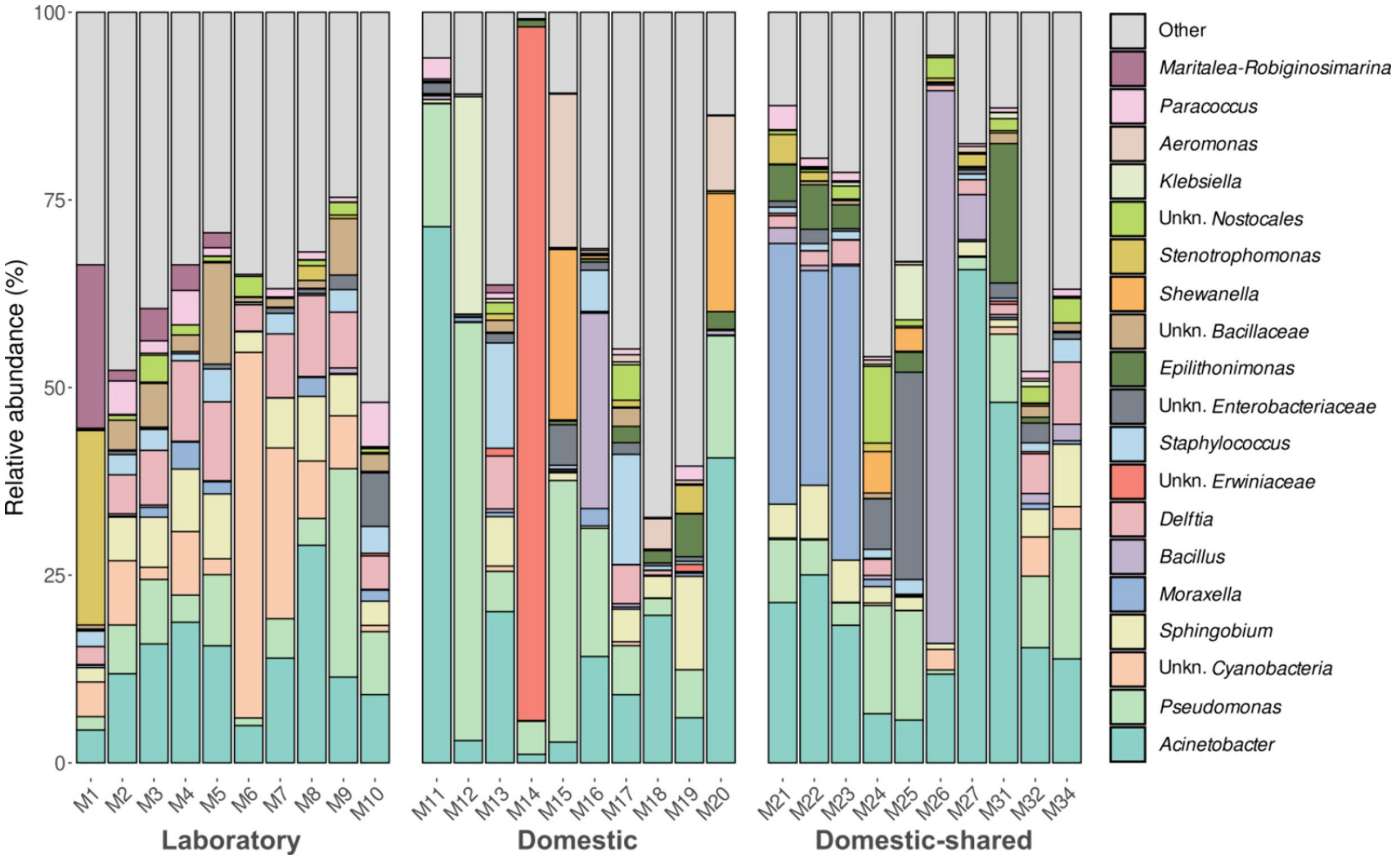
Taxonomic distribution at the genus level of the bacteria present in the three types of microwaves: laboratory (M1–M10), domestic (M11–M20), and domestic-shared (M21–M34).

At the genus level, laboratory microwaves showed a more homogeneous composition than domestic microwaves (Figure 3). *Acinetobacter*, *Pseudomonas*, and *Sphingobium* were present in all types of microwaves. Among the significantly more abundant genera in laboratory microwaves compared to household microwaves were *Delftia*, *Micrococcus*, *Deinocococcus*, and an unidentified genus of the phylum *Cyanobacteria* (Supplementary file S2). The opposite trend was observed for the genera *Epilithonimonas*, *Klebsiella*, *Shewanella*, and *Aeromonas*, among others. In addition, differential abundance analysis between domestic and domestic-shared microwaves showed that two genera, *Lawsonella* and *Methyloversatilis*, were significantly more abundant in the latter group. When comparing NGS results with the culturing techniques, it was found that almost all of the isolated genera were detected by 16S rRNA gene sequencing. Interestingly, *Bhargavaea*, *Janibacter*, and *Nonomuraea*, which could be cultured, were not detected by sequencing.

**Figure 4:**
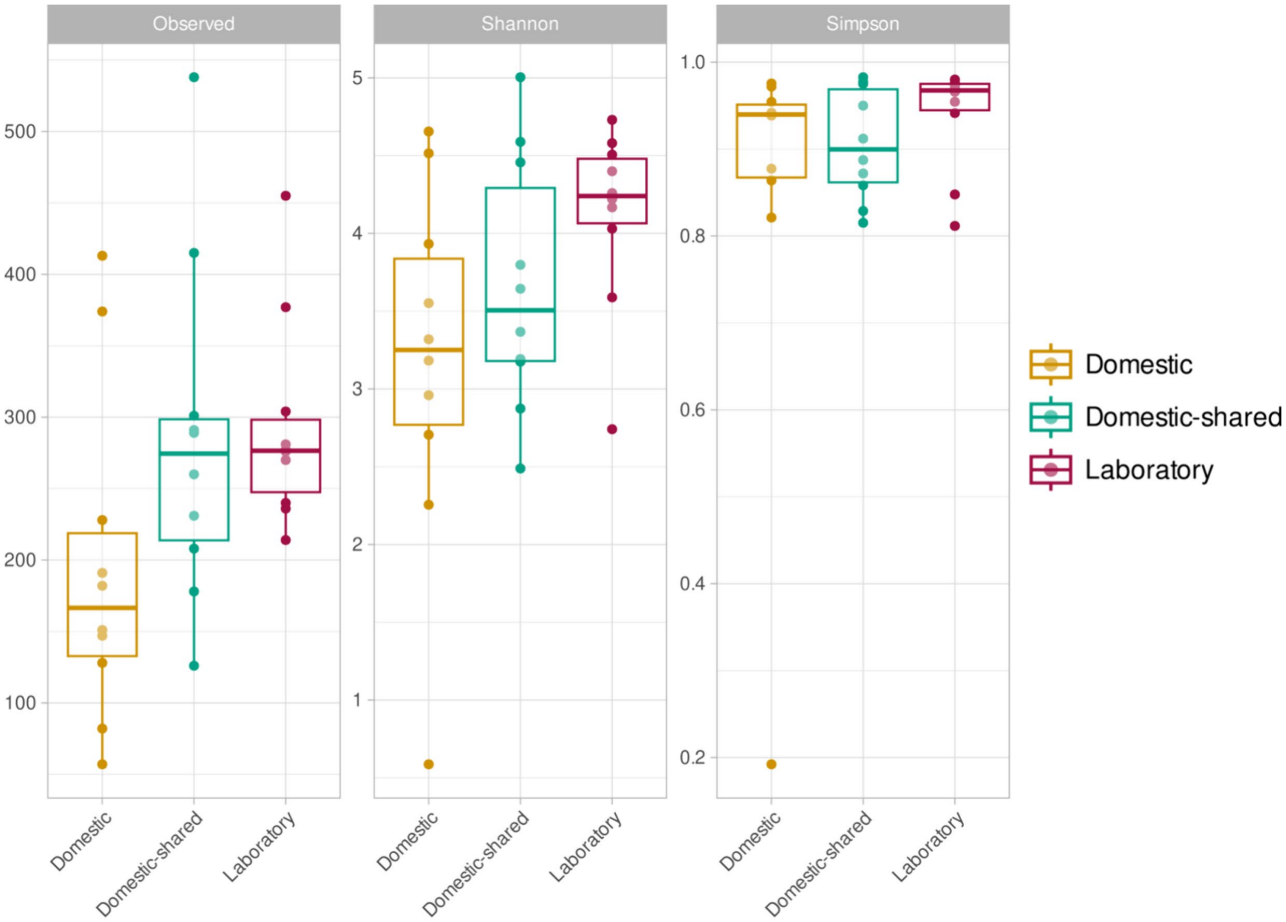
Alpha diversity results (richness or number of ASVs, Shannon index and Simpson index) for the three types of microwaves: laboratory (M1–M10), domestic (M11–M20), and domestic-shared (M21–M34).

In terms of alpha diversity analysis, domestic microwaves had the lowest number of distinct ASVs detected and also lower Shannon index values, although these trends were only significant when comparing this type of sample with laboratory microwaves (Figure 4). No significant differences were found between domestic and domestic-shared microwaves, nor between the latter and laboratory microwaves, in the number of distinct ASVs observed, Shannon index and Simpson index. Overall, between 100 and 300 different ASVs were detected, depending on the type of sample, as well as Shannon indices below 4 in household microwaves and above in laboratory microwaves, while Simpson indices ranged from 0.8 to 1.

**Figure 5:**
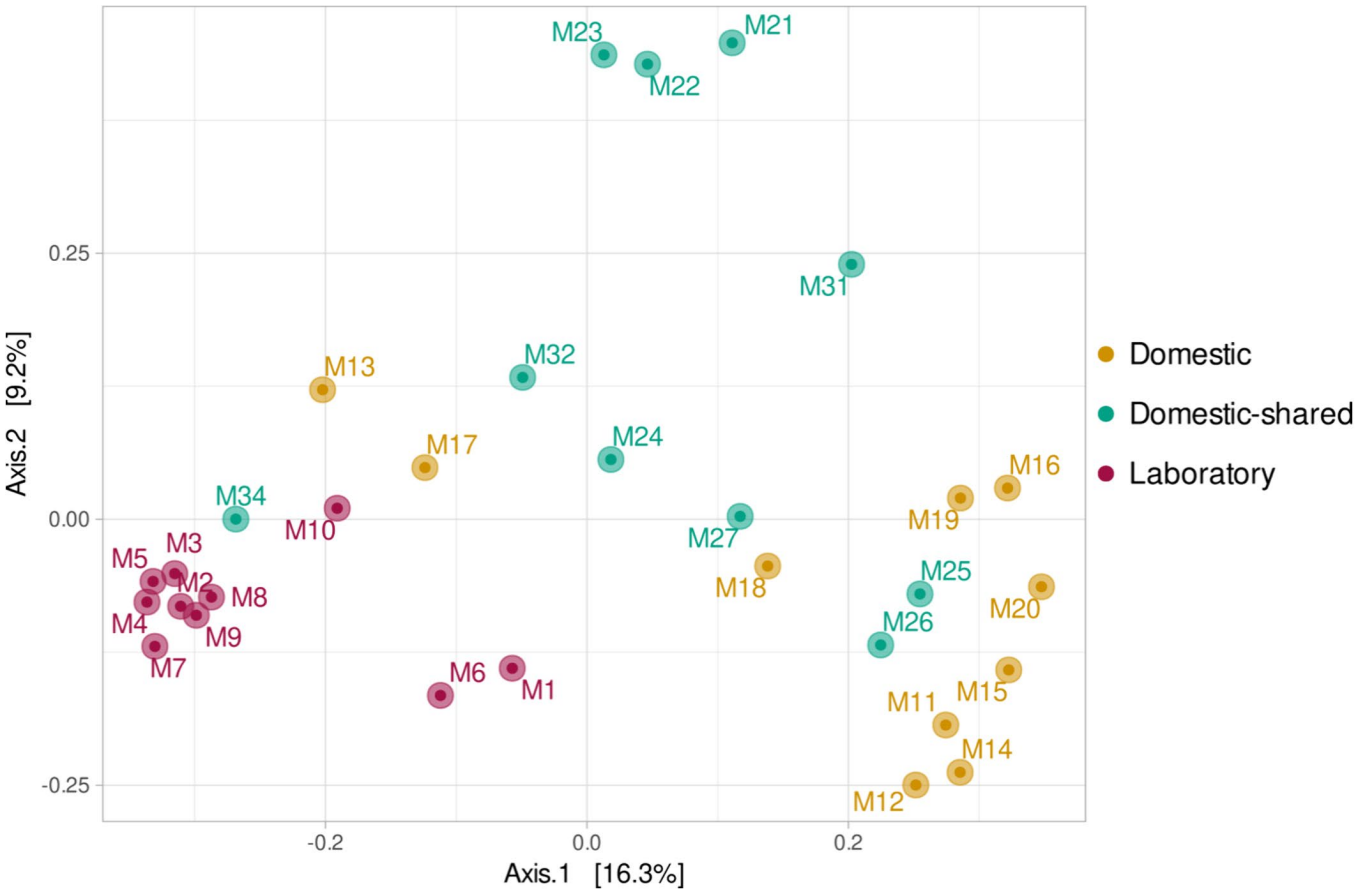
Beta diversity (PCoA) based on Bray–Curtis (ASV level) for the three types of microwaves: laboratory (M1–M10), domestic (M11–M20), and domestic-shared (M21–M34).

**Figure 6:**
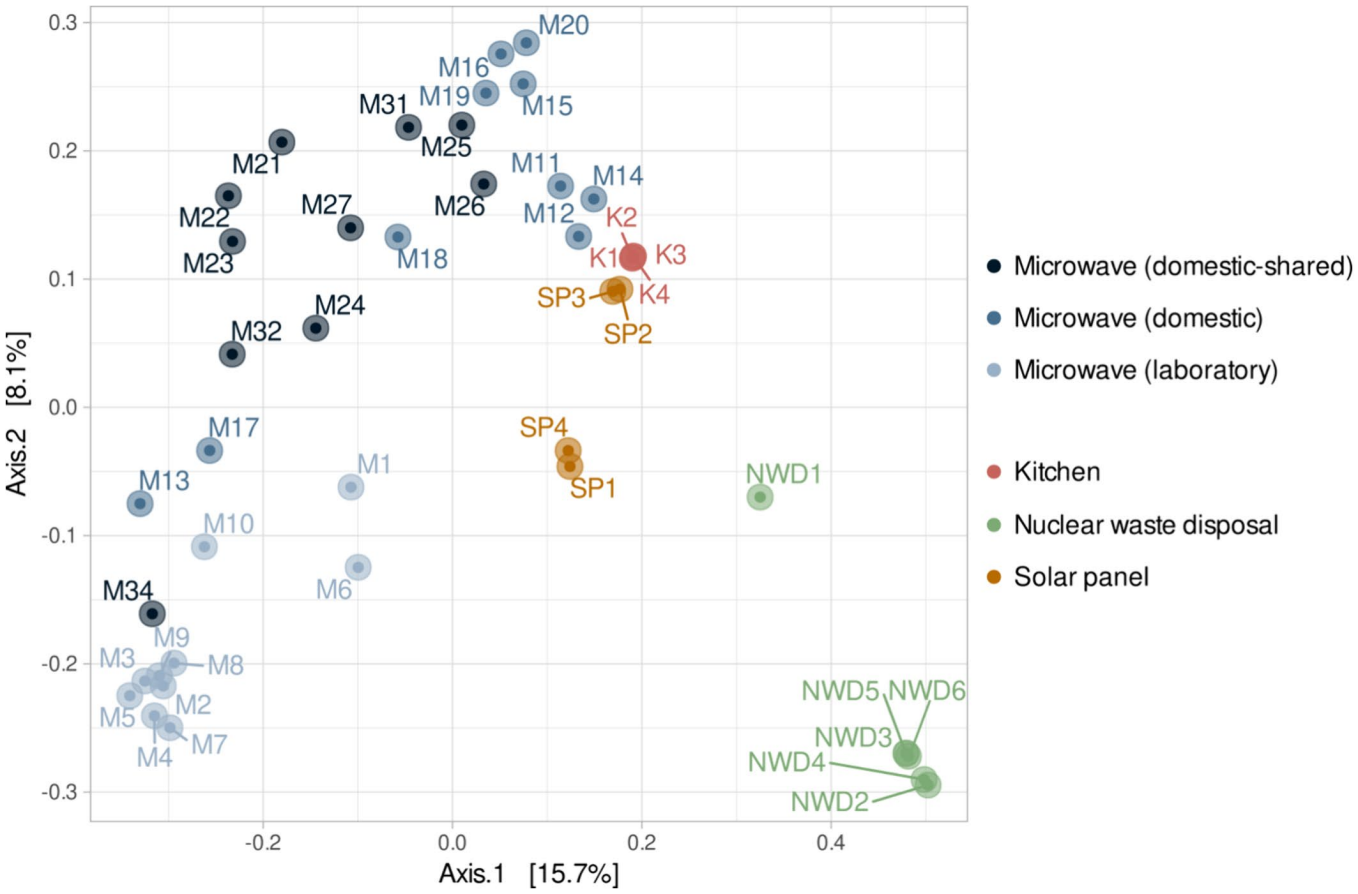
Beta diversity (PCoA) based on Bray–Curtis (ASV level) for the three types of microwaves: laboratory (M1–M10), domestic (M11–M20), and domestic-shared (M21–M34) and samples from other studies: four kitchen samples, four samples from solar panels and six from nuclear waste.

At the β-diversity level, when comparing the different groups of samples at a qualitative and quantitative level, it was observed that they were statistically different from each other (PERMANOVA test, *p*-value < 0.05). Laboratory samples grouped closely together, indicating a greater homogeneity in their bacterial composition (Figure 5). When comparing household microwaves, samples tended to cluster within each of the two groups (domestic and shared-domestic), although this was less evident than with laboratory microwaves. Furthermore, the β-diversity of the microwave samples was also compared with that of two highly irradiated, extreme environments: solar panels and nuclear waste samples; as well as an anthropized indoor environment: kitchen surfaces (Figure 6). The samples were grouped according to their origin, although the solar panel samples and especially the kitchen samples appeared to display a more similar bacterial composition to the household microwave samples. The nuclear waste disposal samples showed the least similarity to the microwave samples.

## 3 Discussion

In this study, we describe the bacterial communities of microwaves by NGS and compare the results obtained in domestic microwaves, domestic use microwaves located in large shared spaces, and laboratory microwaves. In parallel, this work was complemented with the isolation of culturable microorganisms from the same samples.

Through culturing techniques, we found that many of the isolated strains belonged to typically commensal and anthropic genera such as *Bacillus*, *Micrococcus*, *Staphylococcus*, *Micrococcus*, and *Brachybacterium* (Moskovicz et al., 2021; Skowron et al., 2021; Boxberger et al., 2022). As might be expected, human skin-related microorganisms are often found on artificial devices with which humans have frequent contact (Fujiyoshi et al., 2017). In addition, strains belonging to genera potentially pathogenic to humans, such as *Klebsiella* or *Brevundimonas*, were identified in some samples (Podschun and Ullmann, 1998; Ryan and Pembroke, 2018). Although these genera are less common on the skin, they can be found in the human microbiome on mucosal surfaces (Paczosa and Mecsas, 2016; Leung et al., 2019).

Analysis of the 16S rRNA gene revealed that the bacterial communities of the microwaves were dominated by members of the phyla *Proteobacteria*, *Firmicutes*, *Actinobacteria*, and *Bacteroidetes*, which also correspond to the predominant phyla in the human skin microbiome (Cho and Blaser, 2012), serving as an indicator of microwave anthropization. In this regard, the relevant presence of taxa that can be found in human skin such as *Acinetobacter*, *Pseudomonas*, *Moraxella, Bacillus*, and *Staphylococcus* (Kumar et al., 2019) was also detected at the genus level. Despite the similarities found between the samples due to the frequent use of microwaves by humans, differences were also detected between the three types of microwaves, especially between laboratory and domestic microwaves. In the latter, an enrichment of food-associated genera was anticipated due to their primary culinary application. Consequently, it was logical to observe more abundant genera such as *Shewanella*, *Enterobacter*, *Aeromonas*, *Lactococcus*, or *Klebsiella* in this type of microwaves, as they are frequently detected in food matrices and food-related habitats, typically associated with degradation or spoilage processes (Jarvis et al., 2018). It is important to note that certain species belonging to some of these genera, such as *A. hydrophila*, *K. pneumoniae*, and *E. cloacae*, are common contaminants in various food-related habitats and they pose potential health risks due to their pathogenic properties and antibiotic resistance (Daskalov, 2006; Shaker et al., 2007; Rodrigues et al., 2022). Their presence in the microwaves, as well as on other surfaces in the built environment, suggests the importance of regular cleaning practices to mitigate potential health risks, as frequent and adequate cleaning with appropriate disinfectants helps to prevent the presence of pathogens associated with these domestic environments (Carstens et al., 2022). As for laboratory microwaves, their use is completely different, as they are never used to heat food, but mainly to heat aqueous solutions, biological samples, synthetic materials or chemical reagents. Since food cannot be a shaping factor of their microbiomes, we hypothesize that the primary factor determining the microbiome in laboratory microwaves is the extreme conditions created within them (with heating processes that often require longer exposure times). In fact, some of the genera that were significantly more abundant in this group of samples included species known for their resistance to high doses of radiation, such as *Deinococcus*, *Hymenobacter*, *Kineococcus*, *Sphingomonas*, and *Cellulomonas* (Nayak et al., 2021). Some of the mechanisms used by bacteria to withstand such adverse conditions include expression of heat shock proteins (HSPs) (Maleki et al., 2016) and antioxidant enzymes (Munteanu et al., 2015), maintenance of cell integrity through changes in membrane fatty acid composition (Chen and Gänzle, 2016), biofilm formation (Bogino et al., 2013), or DNA repair (Sghaier et al., 2008). In particular, *Deinococcus* species such as *D. radiodurans* and *D. geothermalis* are known for their ability to withstand extreme environmental conditions such as ionizing radiation, desiccation, or high temperatures due to their highly efficient DNA repair mechanisms and protective cellular components (Mattimore and Battista, 1996; Liedert et al., 2012). Moreover, a previous study by Shen et al. (2020) showed that *Acidovorax* and *Aquabacterium*, two other genera enriched in laboratory samples, were differentially more abundant than others at higher temperatures. The phylum *Cyanobacteria* and *Chloroflexi*, which were also more common in laboratory microwaves, have also been described as extremophiles that can withstand environments with high levels of radiation and temperature (Lacap et al., 2011; Uribe-Lorío et al., 2019). The greater presence of bacteria resistant to these types of selective pressures could explain the higher alpha diversity values found in laboratory versus domestic microwaves. In addition, the more frequent use of domestic-shared microwaves and by more people could also favor greater diversity in this group with respect to domestic microwaves, as seen in other devices like washing machines (Jacksch et al., 2021).

In addition, when the bacterial communities of microwaves were compared with those of other highly irradiated environments—solar panels and nuclear waste residues—and kitchens (food-related habitats in constant contact with humans), it was found that domestic microwaves were more similar to kitchen surface samples. However, laboratory microwaves appeared to have similarities to kitchen and, to a lesser extent, solar panel samples. Thus, genera such as *Acinetobacter*, *Pseudomonas*, *Bacillus*, and *Staphylococcus*, widely present in the vast majority of microwaves analyzed, are typical of kitchens (Speirs et al., 1995; Malta et al., 2020). Interestingly, many of the genera significantly more present in laboratory microwaves (such as *Deinococcus*, *Hymenobacter*, *Sphingomonas*, *Ralstonia*, or *Micrococcus*) are typically identified in solar panels (Porcar et al., 2018; Tanner et al., 2018, 2020). These results confirm that all microwave samples resembled each other, although the laboratory microwaves showed greater similarities with microbiomes from environments with relatively low organic matter and subjected to intense radiation or desiccation.

Further work is needed to study the microbial adaptations of strains isolated from microwaves to high temperatures, desiccation, and electromagnetic radiation. For example, although the ability of bacteria to tolerate high temperatures can greatly vary depending on species and strains, those present in higher abundance in microwaves -*Acinetobacter*, *Pseudomonas*, *Delftia*, *Bacillus*, and *Sphingobium*- are known to exhibit a range of tolerance to high temperatures, where *Acinetobacter* has been reported to tolerate up to 50°C (Hrenovic et al., 2014), *Pseudomonas* up to 45°C (Silby et al., 2009), *Delftia* up to 40°C (Roy and Roy, 2019), *Bacillus* up to 80°C (Thomas, 2012) and *Sphingobium* up to 40°C (Singh et al., 2023). Some strains of *Acinetobacter* and *Pseudomonas* have been found to survive for extended periods of time in dry environments, including hospital surfaces (Espinal et al., 2012) and air filters (Pinna et al., 2009) respectively, while *Bacillus* species are well-known for their ability to form spores that can survive in a desiccated state for many years (Checinska et al., 2015). Similarly, some species of *Sphingobium* have been found to survive in dry soil and sediment environments (Madueño et al., 2018).

## 4 Conclusion

Three types of microwaves were studied in order to shed light on their bacterial communities. Our findings revealed the intricate interplay between microwave radiation exposure, food interactions, and user habits in shaping the bacteriome of microwaves. The distinct microbial composition observed between laboratory and household microwaves underscored the influence of usage patterns on microbial communities. Household microwaves, enriched in food-associated genera, reflected their primary culinary use, while laboratory microwaves harbored radiation-, desiccation-, and high-temperature-resistant taxa, indicating prolonged exposure to microwave radiation and suggesting a selective pressure of such harsh factors in shaping the distinctive microbial profile we found. However, more research is needed to understand how certain bacterial strains commonly found in microwaves adapt to these selective pressures. Indeed, this analysis could provide relevant information regarding the biotechnological potential of the microwave bacteriome.

## 5 Experimental procedures

### 5.1 Sampling

The inner cubicle of 10 domestic, 10 shared-domestic and 10 laboratory microwaves was sampled by rubbing a sterile collection swab humidified with Phosphate Buffer Saline solution (PBS, composition in g l-1: NaCl; 8.0, KCl; 2.0, Na_2_HPO_4_; 1.44, KH_2_PO_4_; 0.24. pH; 7.4) that was stored in Eppendorf tubes containing 500 μL PBS and transported to the laboratory at ambient temperature (20–25°C). Samples were immediately used for strain isolation and stored at −20°C until genomic DNA was extracted. A detailed list of the samples taken, and the corresponding microwaves characteristics can be found in Supplementary Table S1.

### 5.2 Strain isolation and identification

For bacterial isolation through culturing techniques, five different growth media were used in this study: Nutrient Agar (NA, composition in g/L: peptone 5, meat extract 3, NaCl 5, agar 15, pH 7.2), Reasoner’s 2A agar (R2A, composition in g/L: peptone 1, yeast extract 0.5, dextrose 0.5, soluble starch 0.5, K_2_HPO_4_ 0.3, MgSO_4_ 0.05, sodium pyruvate 0.3, 15 agar, pH 7.2), Trypticase Soy Agar medium (TSA, contained in g/L: tryptone 15, soya peptone 5, NaCl 5, agar 15, pH 7.2), Yeast Mold Agar medium (YM, contained in g/L: yeast extract 3, malt extract 3, dextrose 10, peptone soybean 4, agar 15, pH; 6.2), Columbia Blood Agar medium (CBA, contained in g/L: special peptone 23, starch 1, NaCl 5, agar 10, pH 7.3).

Samples were homogenized in Eppendorf tubes by vigorously mixing with a vortex, and serial dilutions were plated on the media above and incubated at room temperature for 7 days. After 1 week of incubation, individual colonies were selected and isolated by re-streaking onto fresh medium. Pure cultures were cryo-preserved at −80°C in 15% glycerol.

For the taxonomic identification of the strains, PCRs amplifying a fragment of the 16S rRNA gene were carried out using the universal primers 8F (5′-AGA GTT TGA TCC TGG CTC AG-3′) and 1492R (5′-CGG TTA CCT TGT TAC GAC TT-3′) after extracting the DNA by boiling the cells at 99°C for 10 min in MilliQ-water. The 16S rRNA PCR was performed using the NZYTaq II 2× Green Master Mix, and the following PCR cycle: initial denaturation at 95°C for 3 min; 30 cycles of amplification (15 s at 94°C, 15 s at 50°C, 50 s at 72°C); and 2 min of extension at 72°C. The PCR products were checked by electrophoresis in a 1.2% agarose gel and subsequently precipitated overnight in isopropanol 1:1 (vol:vol) and potassium acetate 1:10 (vol:vol; 3 M, pH 5). DNA pellets were washed with 70% ethanol, resuspended in 15 μL Milli-Q water and Sanger sequenced by Eurofins Genomics (Germany). All the sequences were manually trimmed before comparing them against the EzBioCloud^1^ and NCBI online databases.^2^ EzBioCloud was used to taxonomically identify the closest type strains.

### 5.3 Isolation of genomic DNA

Genomic DNA was isolated from the samples using the PowerSoil DNA Isolation kit (MO BIO laboratories, Carlsbad, CA, United States) following the manufacturer’s instructions and quantified using the Qubit dsDNA HS Assay kit (Qubit 2.0 Fluorometer, Q32866). Three DNA extractions of new, unused sterile collection swabs humidified with PBS solution were also carried out, one of them together with the microwave’s samples and the remaining two on different subsequent days. These two later ones were sent for high-throughput rRNA sequencing separately in two other sequencing batches with samples belonging to other projects.

### 5.4 High-throughput rRNA sequencing and metataxonomic analysis

In order to study the bacterial communities present in the microwaves, the extracted genomic DNA was used to amplify the hypervariable region V3-V4 of the 16S ribosomal RNA gene. The conserved regions V3 and V4 (459 bp) of the 16S rRNA gene were amplified using the following forward and reverse primers: 5′-TCG TCG GCA GCG TCA GAT GTG TAT AAG AGA CAG CCT ACG GGN GGC WGC AG 3′ and 5′-GTC TCG TGG GCT CGG AGA TGT GTA TAA GAG ACA GGA CTA CHV GGG TAT CTA ATC C-3′, and the following PCR cycle: initial denaturation at 95°C for 3 min; 25 cycles of amplification (30 s at 95°C, 30 s at 55°C, 30 s at 72°C); and 5 min of extension at 72°C (Satari et al., 2020). The amplification was carried out using the KAPA HiFi HotStart ReadyMix PCR kit (KK2602). The 16S rRNA amplicons were mixed with Illumina sequencing barcoded adaptors (Nextera XT index kit v2, FC-131-2001), and libraries were normalized and merged. The pools with indexed amplicons were loaded onto the MiSeq reagent cartridge v3 (MS-102-3003) and spiked with 10% PhiX control to improve the sequencing quality, that was finally conducted using paired-ends on an Illumina MiSeq platform (2 × 300 bp) in the Foundation for the Promotion of Health and Biomedical Research of the Valencian Community (Fisabio) (Valencia, Spain).

The raw Illumina sequences were loaded into Qiime2 (v2021.2.0) (Bolyen et al., 2019). The quality of the sequences was checked using the plugin Demux and the Qiime2-integrated DADA2 pipeline was used for trimming and joining the sequences, removing chimeras and detecting amplicon sequence variants (ASVs) (>99.9% of similarity). The taxonomy of each sequence variant was determined via the classify-Sklearn module from the feature-classifier plugin, employing Greengenes-SILVA-RDP (GSR) (Molano et al., 2024) as reference database for the 16S rRNA taxonomic assignment (V3-V4 hypervariable region). Results were analyzed and plotted with the phyloseq R package (v. 1.30.0) (McMurdie and Holmes, 2013) and ggplot2 (v3.4.0).

The beta diversity analysis was carried out using the principal component analysis (PCoA) after calculating the distances between samples using the Bray-Curtis method, using phyloseq R package (v. 1.22.3) (McMurdie and Holmes, 2013) with Bray–Curtis dissimilarities. PERMANOVA tests were calculated with vegan using the adonis2 function from the vegan R package (v2.6.4) to detect statistically significant differences in the composition of the microbiome between the groups analyzed. The differential abundance analyses between taxa were conducted using the MaAsLin2 R package (v1.0.0) (Mallick et al., 2021) with the following parameters: min_abundance = 0.01, min_prevalence = 0.33, max_significance = 0.05, normalization = “None,” transform = “LOG,” analysis_method = “LM,” correction = “BH,” standardize = FALSE. Differentially abundant taxa were considered significant if the adjusted *p*-value was less than or equal to 0.05.

Additionally, the bacterial profile obtained in terms of β-diversity was compared with two extreme environments with high levels of radiation: solar panels and nuclear waste samples, along with a human-modified indoor environment represented by kitchen samples (Supplementary Table S2). For this purpose, publicly available datasets were downloaded from NCBI.

## Data availability statement

Raw reads of the samples analyzed in this study are available at NCBI’s Sequence Read Archive (SRA) (Bioproject Accession PRJNA977132).

## Author contributions

AI: Conceptualization, Data curation, Formal analysis, Investigation, Methodology, Supervision, Validation, Visualization, Writing – original draft, Writing – review & editing. LM: Data curation, Formal analysis, Investigation, Methodology, Writing – review & editing. DT: Data curation, Formal analysis, Investigation, Methodology, Visualization, Writing – original draft, Writing – review & editing. MP: Conceptualization, Funding acquisition, Investigation, Project administration, Resources, Supervision, Validation, Writing – original draft, Writing – review & editing.

## Footnotes

^1^ https://www.ezbiocloud.net

^2^ https://blast.ncbi.nlm.nih.gov/Blast.cgi
