# Supplementary material for: The microwave bacteriome: biodiversity of domestic and laboratory microwave ovens
Source: Front Microbiol. 2024 Aug 8;15:1395751. doi: 10.3389/fmicb.2024.1395751 (PMC11338789; doi:10.3389/fmicb.2024.1395751)
Supplement: Supplementary file 1 [file Data_Sheet_1.zip › Supplementary Material.docx]

***Supplementary Material***

## **Supplementary Figures**


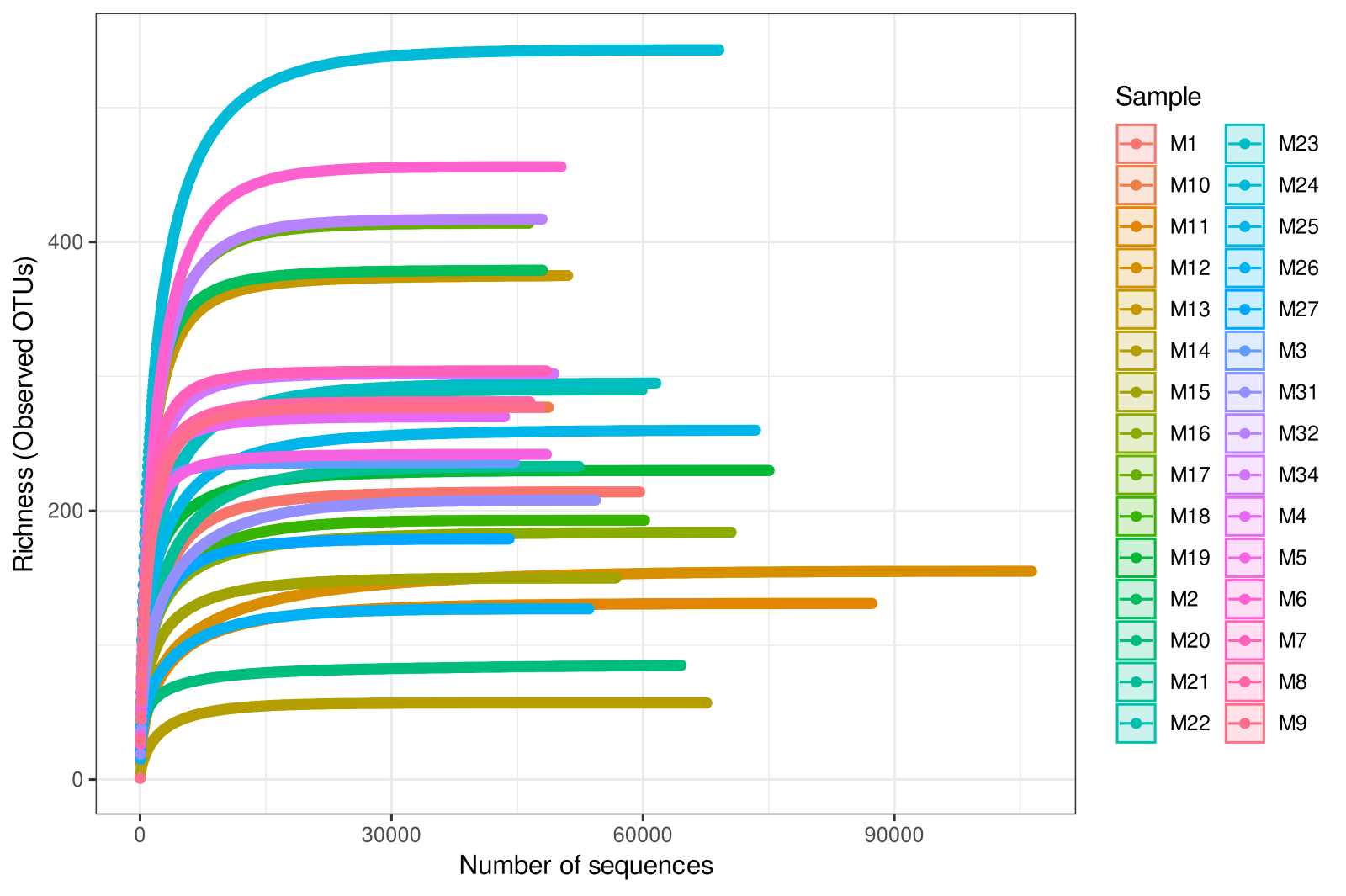


**Figure S1.** Rarefaction curves of all microwave samples.

**Supplementary Tables**

**Table S1.** Microwaves sampled, main characteristics and available usage data.

| **Microwave ID** | **Use** | **Brand** | **Model** | **Minimum and maximum power (W)** | **Most frequently used power (W)** | **Cleaning regularity** | **Last cleaning before sampling** | **Periodicity of use on average** |
| --- | --- | --- | --- | --- | --- | --- | --- | --- |
| M1 | Laboratory | LG | MS-197H | 70-1000 | 1000 | Unknown | Unknown | Daily |
| M2 |  | LG | MS-197H | 70-1000 | 1000 | Unknown | Unknown | Daily |
| M3 |  | Candy | cmxg25gdss | 900-1450 | 1450 | Unknown | Unknown | 3 times per week |
| M4 |  | LG | Wavedom | 90-1000 | 1000 | Unknown | Unknown | Unknown |
| M5 |  | Samsung | mg23f301tak | 800-2300 | Unknown | Every 3 months | 1 week | 3 times per week |
| M6 |  | Jens | wp-750J | 750-1180 | 1180 | Unknown | Unknown | Unknown |
| M7 |  | Moulinex | 1m800pp00 | 800 | 800 | Unknown | Unknown | Unknown |
| M8 |  | Sanyo | em-g4750 | 1200-2600 | Unknown | Unknown | 3 weeks | Max 2 times per week |
| M9 |  | Micrologic | dmr-801 | 230-900 | 900 | Every 3 months | 3 weeks | Daily |
| M10 |  | Whirlpool | mwf421sl | 220-1350 | Unknown | Unknown | Unknown | Daily |
| M11 | Domestic | Daewoo | kog-2q17 | Unknown | max | Every 3 months | 3 months | Daily |
| M12 |  | Daewoo | k06-8a6r | 800-2200 | 1200 | Weekly | 1 week | Daily |
| M13 |  | Aspes | amw120b | baja-alta | alta | Weekly | 1 week | Daily |
| M14 |  | Siemens | hf2565l2 | 90-1220 | 1220 | Every 2 – 4 weeks | 2 days | Daily |
| M15 |  | Samsung | GW71B | 100-750 | 750 | Every 2 – 4 weeks | 1 week | Daily |
| M16 |  | LOGIK | L20MS14 | 1200 | 1270 | Every 2 – 4 weeks | 2 weeks | Daily |
| M17 |  | Unknown | Unknown | Unknown | Unknown | Unknown | Unknown | Unknown |
| M18 |  | Smeg | fme20ex.2 | 850 | 850 | Weekly | 2 days | Daily |
| M19 |  | TEKA | tmw1881 | Unknown | Unknown | Every 2 – 4 weeks | 1 week | Daily |
| M20 |  | Severin | mw7857 | 120-700 | 700 | Daily | 1 day | Daily |
| M21 | Shared domestic | Jocel | jmo011459 | 700-1150 | Unknown | Unknown | Unknown | Unknown |
| M22 |  | Fagor | mwo-20dgw 956010040 | 800-1270 | Unknown | Unknown | Unknown | Daily |
| M23 |  | Orbegozo | mi2015 | 700-1200 | Unknown | Unknown | Unknown | Daily |
| M24 |  | TEKA | MWE 204 FI | Unknown | Unknown | Unknown | Unknown | Daily |
| M25 |  | HORECA | GMW1025 | 1000-1500 | Unknown | Daily | Unknown | Daily |
| M26 |  | Samsung | cm1098a | 1050-1600 | Unknown | Daily | Unknown | Daily |
| M27 |  | Samsung | cm1098a | 1050-1600 | Unknown | Daily | Unknown | Daily |
| M31 |  | Cecotec 13100 | 1522 | 1150-700 | 1150 | Unknown | Unknown | Daily |
| M32 |  | Cecotec 13100 | 1522 | 1150-700 | 1150 | Unknown | Unknown | Daily |
| M34 |  | Sammic | HM-910 | 900-1200 | Unknown | Daily | Unknown | Daily |

**Table S2.** Summary of publicly available 16S rRNA raw reads from solar panels, nuclear waste dumps and kitchens downloaded for comparison with microwave samples in terms of β-diversity.

| **Sample ID** | **Sample Type** | **BioProject** | **BioSample** | **Run** | **Location** | **Reference** |
| --- | --- | --- | --- | --- | --- | --- |
| SP1 | Solar panel | PRJNA806867 | SAMN25936690 | SRR18015359 | Paterna (Valencia, Spain) | Tanner *et al*., 2020 |
| SP2 | Solar panel | PRJNA806867 | SAMN25936689 | SRR18015360 | Paterna (Valencia, Spain) | Tanner *et al*., 2020 |
| SP3 | Solar panel | PRJNA806867 | SAMN25936688 | SRR18015361 | Paterna (Valencia, Spain) | Tanner *et al*., 2020 |
| SP4 | Solar panel | PRJNA806867 | SAMN25936687 | SRR18015362 | Paterna (Valencia, Spain) | Tanner *et al*., 2020 |
| NWD1 | Nuclear waste disposal | PRJNA559268 | SAMN12530750 | SRR9929930 | Buxton (Derbyshire, England) | https://bit.ly/3Kj25mX |
| NWD2 | Nuclear waste disposal | PRJNA559268 | SAMN12530751 | SRR9929931 | Buxton (Derbyshire, England) | https://bit.ly/3Kj25mX |
| NWD3 | Nuclear waste disposal | PRJNA559268 | SAMN12530752 | SRR9929932 | Buxton (Derbyshire, England) | <https://bit.ly/3Kj25mX> |
| NWD4 | Nuclear waste disposal | PRJNA559268 | SAMN12530753 | SRR9929933 | Buxton (Derbyshire, England) | https://bit.ly/3Kj25mX |
| NWD5 | Nuclear waste disposal | PRJNA559268 | SAMN12530754 | SRR9929934 | Buxton (Derbyshire, England) | https://bit.ly/3Kj25mX |
| NWD6 | Nuclear waste disposal | PRJNA559268 | SAMN12530755 | SRR9929935 | Buxton (Derbyshire, England) | https://bit.ly/3Kj25mX |
| K1 | Kitchen (sink) | PRJNA384634 | SAMN07138216 | SRR5574370 | Oakland (California, USA) | Adams *et al*., 2017 |
| K2 | Kitchen (sink) | PRJNA384634 | SAMN07138217 | SRR5574431 | Oakland (California, USA) | Adams *et al*., 2017 |
| K3 | Kitchen (vegetal compartment of refrigerator) | PRJNA415516 | SAMN07828023 | SRR6219629 | Guangzhou (Guangdong, China) | Jeon *et al*., 2013 |
| K4 | Kitchen (vegetal compartment of refrigerator) | PRJNA415516 | SAMN07828039 | SRR6219631 | Guangzhou (Guangdong, China) | Jeon *et al*., 2013 |

**Supplementary Material references**

Adams, R. I., Lymperopoulou, D. S., Misztal, P. K., De Cassia Pessotti, R., Behie, S. W., Tian, Y., Goldstein, A. H., Lindow, S. E., Nazaroff, W. W., Taylor, J. W., Traxler, M. F., & Bruns, T. D. (2017). Microbes and associated soluble and volatile chemicals on periodically wet household surfaces. *Microbiome*, *5*(1), 128. <https://doi.org/10.1186/s40168-017-0347-6>

Jeon, Y.-S., Chun, J., & Kim, B.-S. (2013). Identification of household bacterial community and analysis of species shared with human microbiome. *Current Microbiology*, *67*(5), 557–563. <https://doi.org/10.1007/s00284-013-0401-y>

Tanner, K., Molina‐Menor, E., Latorre‐Pérez, A., Vidal‐Verdú, À., Vilanova, C., Peretó, J., & Porcar, M. (2020). Extremophilic microbial communities on photovoltaic panel surfaces: A two‐year study. *Microbial Biotechnology*, *13*(6), 1819–1830. <https://doi.org/10.1111/1751-7915.13620>
